# Ocular Morbidity and Health Seeking Behaviour in Kwara State, Nigeria: Implications for Delivery of Eye Care Services

**DOI:** 10.1371/journal.pone.0104128

**Published:** 2014-08-28

**Authors:** Laura Senyonjo, Robert Lindfield, Abdulraheem Mahmoud, Kahaki Kimani, Safiya Sanda, Elena Schmidt

**Affiliations:** 1 Sightsavers, Haywards Heath, West Sussex, United Kingdom; 2 International Centre for Eye Health, London School of Hygiene and Tropical Medicine, London, United Kingdom; 3 Department of Ophthalmology, University of Ilorin Teaching Hospital, Ilorin, Kwara State, Nigeria; 4 Department of Ophthalmology, University of Nairobi, Nairobi, Kenya; 5 Sightsavers Nigeria, Kaduna, Nigeria; University of Illinois at Chicago, United States of America

## Abstract

**Background:**

There is currently limited information as to which conditions are most prevalent in communities in developing countries. This makes effective planning of eye services difficult.

**Methods:**

3,899 eligible individuals were recruited and examined in a cross-sectional survey in Asa Local Government Area, Nigeria. Those who self-reported an ocular morbidity were also asked about their health-seeking behaviour. Health records of local facilities were reviewed to collect information on those presenting with ocular morbidities.

**Results:**

25.2% (95% CI: 22.0–28.6) had an ocular morbidity in at least one eye. Leading causes were presbyopia and conditions affecting the lens and conjunctiva. The odds of having an ocular morbidity increased with age and lower educational attainment. 10.1% (7.7–13.0) self-reported ocular morbidity; 48.6% (40.4–56.8) of them reported seeking treatment. At the facility level, 344 patients presented with an ocular morbidity over one month, the most common conditions were red (26.3%) or itchy (20.8%) eyes.

**Conclusion:**

Ocular morbidities, including many non vision impairing conditions, were prevalent with a quarter of the population affected. The delivery of eye care services needs to be tailored in order to address this need and ensure delivery in a cost-effective and sustainable manner.

## Introduction

In many low-income countries, eye care services are provided at tertiary and secondary level. Whilst these specialist services are critical for management of complex eye diseases, such as cataract, they are expensive and often concentrated in urban areas, making them inaccessible for much of the population [1]–[3]. As a result, many individuals delay appropriate treatment [4], [5] or seek care from unqualified sources [6], [7].

It is recognised that many eye conditions are amenable to being treated within the community if effective primary care services were available [8]–[10]. This approach is supported by the World Health Organisation and the International Agency for the Prevention of Blindness. However, there is little consensus about whether this is an effective approach to dealing with eye conditions and if so, how they could be delivered in a sustainable and cost-effective way that effectively addresses patient’s needs and does not delay appropriate treatment.

Ocular morbidity is an overarching term that describes any eye disease including both visually-impairing (VICs) and non-visually impairing conditions (NVICs). Many NVICs and mild VICs, for example minor trauma, allergic and infective conjunctivitis, mild refractive errors and presbyopia, whilst not blinding and not requiring specialist treatment by an ophthalmologist, cause distress and result in demand for health services [11], [12].

To decide whether integration of eye care into primary care is possible or desirable, health authorities require a better understanding of (a) the common eye conditions for which individuals seek treatment in their local facilities and (b) the factors determining whether and where such treatment is sought.

This study aimed to address both questions through examining the prevalence and causes of ocular morbidity and eye health seeking behaviour in Asa Local Government Area (LGA) in Kwara state, Nigeria.

## Materials and Methods

### Study design and sampling

A cross-sectional household survey, based on the Rapid Assessment of Avoidable Blindness (RAAB) survey methodology and a prospective survey of health facilities were conducted in Asa LGA, Nigeria.

The household survey followed a two-stage cluster sampling methodology. The primary sampling unit, the enumeration area, was selected using probability proportional to size and compact segment sampling was used to select households. All eligible members of the selected households were included. Eligibility was defined as staying in the household for at least six of the previous twelve months and sleeping in the house either the previous or the following night.

The sample size was calculated based on an estimated ocular morbidity prevalence of 10%, 2% precision, 95% confidence level, 10% allowance for non- response and a design effect of 4 to account for clustering of infective causes of ocular morbidity and the survey sampling design. A total sample size of 3,803 individuals or 39 clusters of 100 individuals (approximately 20 households per cluster) were required.

All 53 health facilities in the LGA were eligible for inclusion in the facility survey. These ranged from health posts and dispensaries to tertiary hospitals. A list of facilities was obtained from the LGA authorities.

### Data collection

The household survey was conducted in June and July 2012 by four teams, each consisting of an ophthalmic nurse, an optometrist and a sociologist. Teams received six days of training. Inter-observer variation was assessed, with all teams required to achieve a kappa score of 0.7 (tested on agreement between diagnostic categories, specifically, conjunctival disease, lens abnormalities and visual acuity).

A household questionnaire, an eye examination questionnaire and a health-seeking behaviour questionnaire were used to collect data.

After written consent from household heads, socio-demographic data and medical ocular histories were recorded for all participants. All members of the household had their presenting visual acuity (VA) measured in each eye using a 6/12 Snellen E optotype for adults and children aged five or older. Younger children were tested using Lea’s Symbols and those under three were assessed using fix and follow.

All participants underwent a basic eye examination with a direct ophthalmoscope. This followed the examination protocol developed by the RAAB methodology. Those with a presenting VA of <6/12 in one or both eyes were tested with a pinhole and when no improvement with pinhole or obvious reason (e.g corneal opacity) was present, also underwent dilated fundoscopy to determine the cause(s) of visual loss. Retinoscopy was carried out in a central location where assessment of best corrected visual acuity was repeated by an optometrist. Testing for presbyopia was conducted outdoors in the best light conditions available.

Individuals aged 35 years and over were asked whether they owned spectacles for near activities. All had near VA tested at 40 cms (with reading glasses for those who had them). The near addition, greater or equal to +1.00 D, needed to improve their near VA to N8 at 40 cms was recorded.

To explore health seeking behaviour each participant was asked whether they had experienced any problem with their eyes in the last month. Those who had reported problems were interviewed about services they used and barriers they experienced when seeking eye care.

In order to reduce non-response bias, survey teams visited each household up to three times, to try and examine any individual initially absent. The survey was also primarily undertaken over the weekends, when individuals were less likely to be away at work or school.

Facility information was collected during August 2012 and data was collected on all patients attending outpatient departments and reporting ocular morbidity as a primary complaint. Information on patient demographics, eye conditions and treatment were recorded using a specifically designed questionnaire.

### Study area

The study was carried out in Asa LGA, Kwara State in western Nigeria. Since 2003, the University of Ilorin teaching hospital and the non-governmental organization, Sightsavers have supported the State Ministry of Health in the delivery of a comprehensive eye programme in Kwara, focusing on the reduction of the prevalence of avoidable blindness. Asa is one of 16 LGAs that comprise Kwara state. The 2006 census reported the total population of Asa LGA as 142,304 [13] and is thought to be representative of the state with respect to demographics and population density.

### Ethics

The study was approved by the Kwara State Ministry of Health Ethical Review Committee and the London School of Hygiene and Tropical Medicine Ethics Committee. After explaining the study, written informed consent was obtained from the head of the household and from each participant. All ocular disorders were either treated in the field or referred to the specialist eye unit in the State capital Ilorin.

### Data analysis

All completed questionnaires were double-entered into an Epi Data 3.0 database. Analysis was performed using STATA 12.0 (StataCorp. 2011. *Stata Statistical Software: Release 12*. College Station, TX: StataCorp LP).

Descriptive statistics were employed to present simple frequencies of the dependent variable and their distribution by sex, age, education and occupation of participants. Chi-squared tests and bi-variable logistic regression were employed to assess variables associated with the prevalence of an ocular morbidity. All factors found to be associated (p values<0.05) were included in a multivariable logistic regression model, and likelihood ratio tests was conducted to determine which risk factors remained associated with an ocular morbidity, after controlling for potential confounding variables.

The household survey data was assumed to be self-weighted but the analysis was adjusted for cluster sampling methodology. Of those who self-reported that they had an ocular morbidity, further descriptive statistics were employed to determine their health seeking behavior, including if they sought treatment, where they went to seek treatment and why they did or didn’t seek care. Chi-square tests were employed to determine if there was an association between where individuals sought treatment and the type of ocular morbidity they reported.

## Results

### Household survey

#### Study participants

A total of 3,899 individuals from 475 households participated (response rate 99.7%). The majority of household heads were male (80.6%; 95% CI: 75.6–84.5), three quarters of them (74.5%; 66.6–81.1) had no schooling and only 30.1% (23.0–38.3) described themselves as literate.

Amongst those examined, 51.7% (49.0–53.3) were female and the median age was 17 years. Of those aged over 15, 64.5% (58.3–70.3) had no education and 64.3% (60.1–68.3) were employed in manual labour.

#### Prevalence of ocular morbidity

A total of 937 individuals (25.2%; 22.0–28.6) had one or more ocular morbidity (including presbyopia or poor visual acuity, defined as being unable to see 6/12), in one or both eyes and potentially at one or more site. The proportion of individuals with more than one ocular morbidity, at different sites, was low (3.7%; 2.9–4.6). The leading cause of an ocular morbidity was presbyopia, which was recorded in 54.1% (50.6–57.6) of those aged 35 or over. The odds of presbyopia increased with age (p<0.001), with 83.3% (75.4–89.1) of those aged 75 or over not being able to see N8 at 40 cm without correction. Only 2.5% of those diagnosed with presbyopia had glasses for near vision.

The proportion of the population over five with poor visual acuity, defined as being unable to see 6/12 unaided was 12.5% (10.3–15.0), with 12.2% of the population unable to see 6/12 in the better eye and still unable to see 6/12 with the pinhole test. Refractive errors in over fives, defined as unable to see 6/12 unaided but could see 6/12 with pinhole or best correction, was present in 2.5% (1.7–3.2) of the population sampled. The majority of under fives, had good visual acuity, with only 0.5% (0.1–2.0) aged 0–3 unable to fix and follow and only 0.8% (0.2–3.4) of children aged 4–5 years unable to see 6/12 using Lea’s symbols.

If presbyopia and poor visual acuity were removed, 16.2% (13.7–19.0), a total of 596 individuals had an ocular morbidity. The main anatomical site was the lens (44.0%; 39.0–49.1) and the conjunctiva (42.6%; 37.9–47.5), Table 1. The primary cause of an ocular morbidity changed with age. In the younger age groups, the main anatomical site for an ocular morbidity was the conjunctiva, whilst in the older age groups (above 65 years of age) the predominant site was the lens.

**Table 1:** Anatomical site of ocular morbidity excluding presbyopia and poor visual acuity.

Site of ocularmorbidity	% of persons with an ocularmorbidity in the site (n = number)	95% CI
Lens	44.0 (262)	39.0–49.1
Conjunctiva	42.6 (254)	37.9–47.5
Optic nerve	15.1 (90)	11.9–18.9
Retina	8.1 (48)	5.8–11.1
Pupil	7.4 (44)	5.5–9.9
Eyelid	5.7 (34)	3.7–8.6
Cornea	4.0 (24)	2.6–6.1
Orbit and globe	2.7 (16)	1.7–4.3

After adjusting for potential confounders, the increased odds of an ocular morbidity were strongly associated with an increase in age (p<0.001) and lower education (p<0.001).

#### Self-reported morbidity

A total of 388, or 10.1% (7.7–13.0) of participants, reported that they had had an ocular morbidity in the last month. Among those identified with an ocular morbidity by the survey team, only 28.8% self-reported an eye problem. The main conditions reported were itchy eyes or poor vision everywhere, Table 2.

**Table 2:** Problems self-reported by individuals.

Problem	% (n = number)	95% CI
Itch in eye	26.6 (103)	20.5–33.6
Poor vision everywhere	20.4 (79)	15.5–26.4
Poor vision in distance	18.8 (73)	13.4–25.8
Red eye	13.1 (51)	9.0–18.8
Pain in eye	8.0 (31)	5.5–11.5
Poor vision close up	7.0 (27)	4.3–11.0
Other	6.2 (24)	4.1–9.3

#### Health-seeking behaviour

Approximately half of those who self-reported an ocular morbidity (48.6%; 40.4–56.8) sought advice or treatment, primarily from a chemist or medical store (a small private facility which sells general medicines) (35.8%; 27.7–44.8), or a general hospital (31.6%; 23.3–41.2). There was an association between the type of problem and where the person sought treatment (p<0.001). Those who self-reported a red eye were more likely to visit a hospital, while those with pain in the eyes were more likely to visit a chemist. Over a third sought treatment within 1 week (39.9%; 30.8–49.8) and a third within 1 month (37.2%; 27.8–47.6). There was little evidence of an association between the type of problem and the timing of seeking care (p = 0.08).

Nearly everyone that sought advice received some treatment (97.4%), mainly eye drops (69.8%; 62.2–76.5) or tablets (9.6%; 22.7–37.6). Among those who responded about the costs of care, 92.9% (86.4–96.5) stated that the treatment was affordable. However, this finding should be treated with caution, as data was missing for over 52% of respondents and as the lack of money was stated as one of the main reasons why someone did not seek care (17.4%; 11.8–24.9). The most common reason for not seeking treatment was a belief that the problem was not important (48.9%; 37.9–60.0). Other key reasons included self-treatment (15.2%; 10.1–22.4) and not knowing where to go (12.0%; 5.9–22.8).

### Health facility data

The data were collected from 45 of the 53 health facilities in LGA, 40 were government facilities and 5 private. This included, 1 general and 1 private hospital, 3 cottage hospitals, 30 health facilities or clinics and 10 dispensaries. Out of 5,549 patients visiting outpatient departments in August 2012, 344 (6.2%) had an ocular morbidity as a primary complaint, although the percentage differed by facility. Just over half, 50.6% of patients were female. About a third of eye patients were children (30.8%), another third (31.4%) were aged 35–54 years and one in five (21.8%) were 55 years and older. The main presenting complaint was red eye (26.3%), followed by itchy eyes (20.8%), and pain (18.2%), Table 3.

**Table 3:** Eye complaints of persons attending outpatient departments.

Complaint	%
Red eye	26.3
Itch	20.8
Pain	18.2
Discharge	9.6
Poor vision everywhere	9.0
Poor vision in the distance	8.3
Poor vision close up	4.6
Other reason	3.3

For the majority (74.7%), this was the first visit to the health facility. Most cases (52.3%) were treated at the clinic, mainly with eye drops (68.0%), 27.6% were referred to a higher level specialist facility.

## Discussion

Health systems in low-income countries have tended to focus on managing blinding conditions and whilst important, this approach may underestimate the burden of an ocular morbidity and its resultant demands on health services. Our study in Asa LGA in Nigeria showed that eye problems, including visually impairing and non-impairing conditions, are common with approximately a quarter of the population having at least one ocular morbidity including presbyopia and poor visual acuity. The results are consistent with earlier surveys in Africa and Asia reporting prevalence between 5.6% and 30% depending on the conditions included [14]–[16].

It appears as though for many individuals, they do not realise that they have an ocular morbidity, as only a quarter of those who had an identified problem diagnosed by the survey team, self-reported an eye problem. Furthermore, among those who recognised they had a problem, only half sought advice or treatment. Although the limitations in the diagnosis in the community make it difficult to accurately determine the percentage of eye conditions that were self-limiting, even if we assume that the majority of eye conditions identified in our survey were self-limiting and did not require treatment, the findings suggest that a high proportion of individuals in this community still missed opportunities for early diagnosis and treatment. This could be because their disease was asymptomatic, they thought it unimportant or they did not know where to access care. The results emphasise a need for further eye health education, raising community awareness and pro-active case finding for conditions that could be potentially blinding, particularly in the populations with high rates of illiteracy as in Asa LGA. Costs of care may be another important barrier to uptake of services, however this issue requires further exploration as over half of our respondents preferred not to answer this question. The capacity to source spectacle correction appeared to be an issue in this area, where presbyopia was the major cause of ocular morbidity and yet only 2.5% of those who could benefit from presbyopic correction had glasses.

There have been some efforts in health promotion activities in Asa LGA, including improving community awareness of key eye conditions and the importance of getting treatment, support to referrals from the community and lower health facilities to specialist eye care services, as well as school eye health programmes. However, the results suggest the uptake of eye services was still not adequate and there may be benefits in reviewing the delivery of current eye care services to ensure they reach those who need the services. However, a better understanding of how people make decisions about seeking care and where they decide to go is necessary for effective planning. It is also important to better understand financial implications of reconfiguring services and to estimate potential financial gains from the health system and individual perspectives.

The majority of people who sought care in our study went to a hospital or a local chemist and there was an association between the type of eye problem and the type of the facility accessed. It appears that more urgent conditions, such as pain in the eyes, presented at a nearby chemist, although there is little information about the quality of services offered there.

The results of the analysis of the facility data are broadly consistent with the survey findings, showing that NVIC and mild VIC are common complaints. An interesting finding was that the majority of patients attending clinics were children and working age adults, suggesting that seeking care may be prioritised in these population groups compared to older people. Another explanation may be that the conditions experienced by these populations (conjunctivitis, trauma) are thought to be acute and therefore more urgent (or treatable) compared to long -term problems experienced by older people due to aging.

A key limitation of the study was using simple examination techniques (e.g. direct ophthalmoscope) to identify conditions, which reduced diagnostic precision for conditions such as glaucoma or conjunctivitis, where more complex tools are required. As a result we described disease using the anatomical site where it occurred instead of a formal diagnosis. This is a similar to the RAAB survey methodology where diagnoses are frequently grouped to limit diagnostic uncertainty. The definition of ocular morbidity was deliberately kept broad without specific diagnostic criteria for each disease the examiners could encounter. This approach was taken for several reasons; to capture the importance of the eye problem from the patient perspective and to keep the survey methods as simple as possible due to the limitations with equipment and techniques employed. The grouping of the diagnostic categories for ocular morbidity was determined by four ophthalmologists and tested through agreement in a formal inter-observer variation test, however there may still be some variation about diagnoses that means that the findings must be interpreted with care.

Further, as we did not collect any qualitative data in this study, we were unable to explore reasons for variations in health seeking behaviour. We also did not assess the type or quality of services provided in the existing facilities and could not examine to what extent the capacity or reputation of a facility itself determined patient choice. Further research is needed to explore these questions.

In conclusion, this study showed that large numbers of people experienced eye conditions in rural Nigeria. However there is a lack of understanding about eye health and the importance of seeking care. When care is sought, patients tend to prioritise local community providers. Providing services close to the communities could reduce distance to care and increase uptake of treatment. It may also free up limited resources at the secondary and tertiary level, allowing for those facilities to focus on more complex eye conditions. However, which services could be better integrated in primary care and how this would be obtained within the current health system structures, needs to be carefully considered [17]–[20]. Further research is required to explore these issues so that the best care is delivered to people in the best location and at the most appropriate time.
