# Correction: Ocular Morbidity and Health Seeking Behaviour in Kwara State, Nigeria: Implications for Delivery of Eye Care Services

**DOI:** 10.1371/journal.pone.0112860

**Published:** 2014-10-31

**Authors:** The PLOS ONE Staff

There are errors in the funding statement. Please see the correct statement below.

This study was funded by Sightsavers. Sightsavers is an international charity that works with partners in developing countries to eliminate avoidable blindness (www.sightsavers.org). The members of the research department (two authors) in Sightsavers had a role in the design, analysis and preparation of this manuscript but did not take part in data collection. One author, a Sightsavers employee and author supported the data collection but was not involved in the study design or analysis.
